# Genome-Scale Metabolic Models in Fungal Pathogens: Past, Present, and Future

**DOI:** 10.3390/ijms251910852

**Published:** 2024-10-09

**Authors:** Angie Lorena Fonseca-Fernández, Andrés Fernando González Barrios, Adriana Marcela Celis Ramírez

**Affiliations:** 1 Grupo de Investigación Celular y Molecular de Microorganismos Patógenos (CeMoP), Department of Biological Science, Faculty of Science, Universidad de los Andes, Bogotá 111711, Colombia; al.fonseca10@uniandes.edu.co; 2 Grupo de Diseño de Productos y Procesos (GDPP), Departament of Chemical and Food Engineering, Faculty of Engineering, Universidad de los Andes, Bogotá 111711, Colombia; andgonza@uniandes.edu.co

**Keywords:** genome-scale metabolic model, fungi, pathogen, virulence

## Abstract

Fungi are diverse organisms with various characteristics and functions. Some play a role in recycling essential elements, such as nitrogen and carbon, while others are utilized in the food and drink production industry. Some others are known to cause diseases in various organisms, including humans. Fungal pathogens cause superficial, subcutaneous, and systemic infections. Consequently, many scientists have focused on studying the factors contributing to the development of human diseases. Therefore, multiple approaches have been assessed to examine the biology of these intriguing organisms. The genome-scale metabolic models (GEMs) have demonstrated many advantages to microbial metabolism studies and the ability to propose novel therapeutic alternatives. Despite significant advancements, much remains to be elucidated regarding the use of this tool for investigating fungal metabolism. This review aims to compile the data provided by the published GEMs of human fungal pathogens. It gives specific examples of the most significant contributions made by these models, examines the advantages and difficulties associated with using such models, and explores the novel approaches suggested to enhance and refine their development.

## 1. Introduction

Fungal-related infections are becoming an increasingly significant global concern. These include superficial, subcutaneous, and invasive fungal diseases (IFDs), which impact a significant percentage of people and have rising rates of morbidity and mortality. Immunocompromised individuals or those with comorbidities such as diabetes, cancer, HIV, tuberculosis (TB), or chronic lung disease are more susceptible to these infections. The rise in these highly vulnerable populations, combined with the increase in antifungal resistance reported in recent years, has prompted the World Health Organization (WHO) to classify some of the microorganisms causing these infections as critical or high-priority pathogens [1]. Indeed, this organization has promoted increased efforts toward several goals, such as promoting international cooperation to develop innovative treatments and diagnostic strategies that improve disease detection, monitoring antifungal resistance acquisition, and training policymakers to design and implement policies focused on preventing antifungal resistance and combating fungal infections [1].

Nonetheless, these efforts have had a limited scope, and antifungal resistance, as well as the limited availability of antifungal treatment alternatives, profoundly affects human health, leading to prolonged therapy periods, extended hospitalizations, and a heightened need for expensive, often highly toxic secondary antifungal treatment [2]. For instance, the critical priority list members, *Aspergillus fumigatus*, *Candida albicans*, and *C. auris*, had showed a growing resistance rate to azole molecules in primarily treated patients with these [1]. In this regard, about 28% of cases had been reported, and Azole-resistant *A. fumigatus* may be found in 3.5% to 5% of clinical isolates in the United States [3] and 9.3% in environmental isolates in Colombia [4]. Besides that, *C. albicans* and non-albicans species like *Nakaseomyces glabratus* (also known as *C. glabrata*), *Pichia kudriavzevii* (also known as *C. krusei*), *C. parapsilosis*, and *C. tropicalis* have become more resistant to antifungals, especially fluconazole [5]. Additionally, *C. auris* is an emergent pathogen that has recently attracted the attention of both the public and the medical community because clinical isolates are resistant to several drugs [3]. Therefore, to address this worldwide health threat, more and new treatment strategies ought to be required in the future.

However, numerous challenges have emerged in this area, including the difficulty of discovering new therapeutic targets due to the similarities between human and fungal cells [6], and the extended time frame and expensive procedures required to approve new antifungal therapies have negatively impacted the effective strategies to deal with this worldwide health problem. As a result, in silico models have drawn interest from academia and the pharmaceutical industry, making it easier to identify novel therapeutic targets and analyze the virulence features of various microorganisms. In this regard, genome-scale metabolic models (GEMs) have emerged as a promising alternative that provides multiple benefits in analyzing pathogenic processes in a wide range of microorganisms [7]. Utilizing GEMs helps us to gain a better understanding of how intracellular infections work by enabling the simulation of numerous infection scenarios in a concise frame of time [8]. They can also be utilized for the identification of novel therapeutic targets, the prediction of compound activity, and the optimization of lead compounds [8,9,10]. Therefore, in this work, we reviewed the fungal pathogen microorganisms’ genome-scale metabolic models (GEMs). The referenced papers have been meticulously chosen according to the modeled microorganisms and their influence on human health. To conduct a comprehensive historical compilation of metabolic models in fungi, we included publications from 2003, which marks the initial publication of these models. The analysis of microbial metabolism includes papers that have produced exceptional results. These papers will significantly enhance our understanding of the biology of fungal pathogens and describe tools that are currently or potentially valuable for the future development of genome-scale metabolic reconstructions. Indeed, we show concrete examples of the fungal pathogen’s available GEMs and discuss their possible applications in developing therapeutic strategies. Additionally, we discuss how the integration of multi-constraints and proteomic, metabolomic, and genomic data in GEMs, as well as the use of machine learning technologies, should help in performing a quantitative analysis not only of pathogen metabolism but also the metabolism involved in the host–pathogen interaction.

## 2. The Overview of GEMs of Fungal Pathogens

Since 1999, when the first genome-scale metabolic model (GEM) for the human pathogen bacteria *Haemophilus influenzae* was published, substantial advancements have been achieved in advancing these models [11]. The GEMs were primarily designed for model organisms, such as *E. coli*, whose GEM was published in 2000. These first models were quite beneficial and established the foundation for further applications of metabolic modeling in various organisms, including fungi [12]. However, the development of new-generation sequencing technologies and the understanding of the genetic traits of other organisms have led to a significant increase in the creation of comprehensive models of microbial metabolism at the genome level [13]. A considerable advancement occurred in 2003 when the first metabolic model of an eukaryotic organism, *Saccharomyces cerevisiae*, was published. This model includes 708 open reading frames (ORFs) in the reconstructed network, resulting in 1175 metabolic reactions and 584 metabolites. However, the reconstructed network includes gene functions for only around 16% of all characterized ORFs in *S. cerevisiae*. Furthermore, as there are no eukaryotic organisms for which GEMs have been published, the metabolic capacities of *S. cerevisiae* were estimated and compared with the GEM of *E. coli* [14]. However, this model provided valuable information about the biology and metabolism of this yeast. Therefore, including the description of the enzymatic reactions and metabolic pathways known at the time, this model allows a deeper understanding of how the different metabolites and pathways interact in the yeast.

Additionally, this model allowed us to calculate the capacity of this microorganism to produce precursor metabolites and the 20 amino acids, starting with glucose as the only carbon source. These results allowed for the postulation of valuable information about the efficiency of this yeast for the industrial production of amino acids, even above *E. coli*. In addition, this model allowed us to simulate phenotypic behavior under different genetic and physiological conditions, which was crucial for understanding how variations in genetics can influence the metabolism and adaptation of the organism [14]. This simulation, although extremely limited compared to those currently available, provided a powerful tool for research in systemic biology, metabolic biology, and genetic engineering, facilitating the understanding of cellular processes and the development of biotechnological applications at the time. In total, about 6000 metabolic models of bacteria and eukaryote organisms have been published [15], and the original models have been improved and revised as additional biological data have become accessible [12], as is described below. Since there is not much information regarding genome-scale metabolic models for fungal pathogens, our attention will be directed towards the available GEMs of fungal pathogens from 2003 to the present (Table 1).

Due to the interest and remarkable progress in these computational techniques, a few years after the first publication of *S. cerevisiae* GEMs, other, less sophisticated studies about the organism’s reactions and metabolites, such as genome-scale network reconstructions (GENREs), were developed. This strategy involves a comprehensive list of the biochemical reactions occurring within an organism, along with specific information regarding cell borders, biomass assembly reactions, and the exchange of fluxes with the external environment [16]. This application was first used on *Neurospora crassa* in 2003 and later expanded to *Aspergillus nidulans*, *A. oryzae*, and *A. fumigatus* in 2005 [13]. This approach contributed to the development of both manual and automated approaches for model reconstruction and network modeling and enabled the identification of promising targets for metabolic engineering before in vivo testing, thereby accelerating the development of industrially important strains to produce acids, antibiotics, and enzymes. Furthermore, GENRE models have been employed to analyze omics data, establishing connections between gene expression information and metabolic fluxes. This has resulted in the identification of novel biological characteristics in these organisms [16]. Later, in 2008, the first *Aspergillus niger* GEM, the iMA871 model, was published. The iMA871 model offers substantial progress in comprehending the metabolic capability of this fungus. After meticulously curating literature data manually, our iMA871 model compiled around 371 articles for bibliomic data, and 871 open reading frames (ORFs), resulting in a metabolic network consisting of 2240 distinct reactions, with approximately 1190 reactions that are exclusive to this network [17]. Moreover, GENRE not only simplifies the identification of targets for metabolic engineering, the comprehension of transcript data, and the analysis of metabolic fluxes, but it also establishes connections between reactions, genes, and existing scientific research, resulting in a comprehensive database on human pathogen metabolism. In addition, the verified outcomes of this model enabled us to identify the significant potential for enhancing the manufacturing of established and innovative products, thereby maximizing the use of *A. niger* as an efficient production platform [17].

Interestingly, the next metabolic reconstruction of a fungal pathogen, *Penicillium chrysogenum*, took approximately five years to be published. In addition, between 2013 and 2019, there were only eight metabolic reconstructions. Among these, two were associated with mold, *Mucor circinelloides* (2016) [18] and *Aspergillus fumigatus* (2019) [19], and six were associated with yeasts. Then, in 2017, the first GEMs of lipid-dependent human pathogen yeasts were published, and these approaches provided evidence of the large differences between *Malassezia*’s species, especially in how they made steroids and use fatty acids, providing crucial information in the knowledge of lipid metabolism in this yeast. In addition, the authors analyzed the distribution of metabolic fluxes related to lipid metabolism across several species, highlighting the extensive versatility of this group of yeasts in terms of their lipid metabolism [20]. These findings highlight the significance of acquiring a more profound understanding of the biology and ecology of *Malassezia*. Indeed, the authors emphasized that conducting omics studies is crucial in understanding the mechanisms involved in the pathogenesis of this microorganism [20].

The genome-scale metabolic models of *Saccharomyces cerevisiae* have experienced substantial enhancements throughout the years. As described above, the initial models, such as iFF708, specifically emphasized the basic description of the yeast metabolism [14]. Years later in 2004, a model called iND750 was published, introducing gene–protein–reaction associations and compartmentalization to the GEM. This model allowed us to classify the yeast’s reactions into eight cellular locations: extracellular space, cytosol, mitochondrion, peroxisome, nucleus, endoplasmic reticulum, Golgi apparatus, and vacuole. Moreover, this enabled the identification of inaccurate predictions, mainly resulting from the model’s insufficient incorporation of biological constraints [21]. The scope of these models was expanded by the models iMH805/775 [22], iMM904 [23], and Yeast1 to Yeast7, resulting in an improved coverage of biochemical features [24], and the more recently developed model Yeast 8, published in 2019, includes 3895 reactions, 2666 metabolites, and 892 genes, as well as notable improvements to its metabolic model. Indeed, additional processes were included to predict growth on similar substrates, revised metabolite annotations, and genes utilized from many databases absent in previous iterations. Furthermore, the biomass equation was revised to incorporate nine trace metal ions and eight cofactors, and 37 transport processes were introduced to eliminate 45 metabolites that were previously unable to proceed. Moreover, the lipid metabolism was restructured using the SLIMEr formalism. These adjustments enhance the scope and prediction capacity of the model and more accurately represent the fundamental metabolic processes of the strains of *S. cerevisiae* [25]. On the other hand, this microorganism has traditionally been recognized as essential in the food and brewing industries; however, some strains have been identified as significant pathogens, primarily impacting those who have had organ transplants or are HIV-positive [26]. Despite the recent reports of these pathogenic isolates and the fact that the metabolism of microorganisms plays a crucial role in their potential virulence, the GEM has not yet been explored as a tool that allows the analysis of pathogenic strains of this yeast. In the future, it will be crucial to utilize GEMs (genome-scale metabolic models) to identify the metabolic factors that promote the diseases caused by this significant microorganism.

**Table 1:** Genome-scale metabolic models of fungal human pathogens.

Organism	Model Names	Number of Metabolites	Number of Reactions	Number of Genes	Compartments	Reference
*Aspergillus niger*	iMA871	1045	2240	871	3	[17]
*Penicillium chrysogenum*		1235	1471	713	4	[27]
*Mucor circinelloides*	*i*WV1213	1413	1326	1213	5	[18]
*Malassezia globosa*		2162	1187	995	5	[20]
*M. sympodialis*		2303	1073	828		
*M. pachydermatis*		1838	1236	1012		
*M. furfur* and atypical *M. furfur*		3103	2188	1853		
		3642	2823	2447		
*Aspergillus fumigatus*			2065			[28]
*Saccharomyces cerevisiae*	iFF708	584	1175	708 *	2	[14]
	iND750	646	1149	750	8	[21]
	iMH805/775	82	775 ^+^	805	4	[22]
	iMM904	1228	1412	904	5	[23]
	Yeast8	2666	3895	892	14	[25]
*Pichia kudriavzevii* (also known as *Candida krusei*)	SD108	1702	1826	850		[29]
*Candida albicans*	iRV781	926	1221	781	4	[30]
*Candida parapsilosis*	iDC1003	1278	1804	1003	5 ^1^	[31]
*Papiliotrema laurentii*	UFV-1	2127	2465	796		[32]
*Candida auris*	iRV973	2150	3546	973	4	[33]
*Cryptococcus neoformans*	iCryptococcus	1143	1270	649	8	[34]

^1^ This GEM has four compartments and an intercompartment: the cytoplasmic membrane. * indicates structural open reading frames (ORFs). ^+^ indicates regulatory interactions.

Subsequently, as more genomic data and powerful bioinformatics tools became available, metabolic models of pathogenic fungi became available. Between 2020 and 2023, researchers published GEMs of *C. albicans* [30], *C. parapsilopsis* [31], *Pichia kudriavzevii* (also known as *Issatchenkia orientalis* and *Candida krusei*) [29], and *C. auris* [33]. One of the key discoveries of these models was the identification of crucial enzymes necessary to reproduce and adapt to the host condition. Moreover, the metabolic evidence of those models opens the possibility of new therapeutic targets [30]. One of the most prominent examples is *C. auris* due to its relevance as a multi-resistant human pathogen. This pathogen’s GEM has allowed us to identify unique enzymes in this yeast, even when we compare it to other yeasts of the same genus. For instance, the quinate dehydrogenase (EC 1.1.1.24) converts quinate into a building block for producing folates, quinones, and aromatic amino acids. Another example is the enzyme chloride peroxidase (EC 1.1.1.10), which aids in producing hypochloric acid by transferring an oxygen atom from H_2_O_2_ to chloride [33]. These two enzymes could be related to the defense mechanism of *C. auris* against its host and have been proposed as powerful alternative targets against this important pathogen. Furthermore, this GEM proposes about 82 unique genes related to steroid biosynthesis, purine and pyrimidine metabolism, ergosterol biosynthesis, or chitin production, such as *Erg4* and *Chs4*, whose human ortholog genes have not been identified, highlighting them as potential therapeutic targets too [33]. However, despite the lack of experimental validation, these genes, which have no identified homologs in humans, are very promising candidates for developing new drugs. Such forward experimental validations will be crucial to ensure that genome-wide metabolic models (GEMs) accurately reflect real biology, thereby increasing their clinical applicability by confirming the efficacy of therapeutic targets in treating these infections [35].

Later, the GEM of *C. neoformans* was published. This model significantly contributed to studying the host–pathogen relationship between *C. neoformans* and human macrophages. Interestingly, the intracellular environment of the macrophage is defined by a reduced glucose content, and these conditions affect the host’s colonization. Thus, this metabolic model infers that the microorganism can activate another metabolic pathway as an alternative carbon source when exposed to low glucose concentrations. Moreover, it is known that one of the most important structures involved in the virulence of this yeast is the capsule [34]. Therefore, this GEM proposes a metabolic pathaway for capsule synthesis. Consequently, UDP-glucuronate was named as the main source of capsule structure parts needed to produce alpha and beta-glucans. Despite the final steps of the capsule formation remaining unclear, this pathway reconstruction opens the possibility of studying the enzyme needed to form this virulence factor as another therapeutic target against this opportunistic pathogen [34]. These results highlight the important role of GEM development in both industrial and clinical fields, providing essential knowledge about metabolism, the classification of strains, and the virulence and adaptation of species. Nevertheless, it is necessary to increase the use of these models to explore further aspects of the biology of diverse fungal pathogens, including their virulence factors.

Historically, the progress in metabolic reconstructions of fungal infections has been restricted due to technical difficulties and insufficient biological understanding. However, these methods have become more significant due to technological advancements and an increased understanding of the biological systems under investigation. In the subsequent part, we examine specific instances of how these reconstructions are currently being implemented in metabolic analysis, along with the constraints that hinder their implementation and efficacy. This will underscore the necessity of ongoing innovation in this domain.

## 3. The Present: Advantages and Limitations of GEM

**Figure 1:**
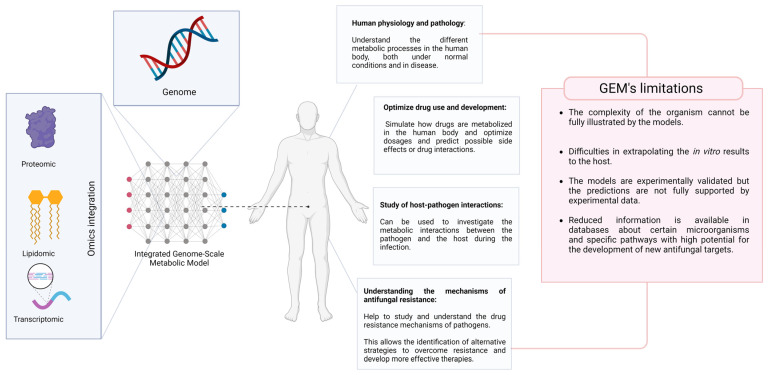
Illustration of basic genome-scale metabolic models’ components: the genome annotation and omics integration to study the fungal human pathogen and its potential advantage to human health (in blue blocks) and its limitation (in red blocks). Created with BioRender.com [42].

Metabolic models offer a unique approach to studying human infections, providing insights into pathogenicity, resistance, and host–pathogen interaction [8,36]. While the application of this technique to fungi is currently restricted, it is gradually gaining popularity; therefore, certain limitations of these models for metabolic research have been recognized. Hence, we discuss, with specific examples, the more interesting and significant advantages and limitations of existing models about fungal human pathogens. Figure 1 illustrates the potential applications of the GEM in the virulence and resistance analysis of microorganisms, as well as the limitations identified in this process.

Among the more attractive topics of microbial nature is the emergence of antifungal resistance, which has been described as a global public health concern in recent years. There have been numerous cases where pathogens have developed resistance to antifungal molecules previously employed as treatments. Therefore, when analyzing a microorganism’s role in disease development, assessing its ability to acquire and transfer resistance mechanisms that can significantly impact therapeutic outcomes is essential. A notable example of that is *C. auris*, a yeast discovered in Japan in 2009 and for which the WHO has elevated it to the status of a critical-priority microbe [37] due to its remarkable resistance to several antifungal drugs and its propensity to cause hospital outbreaks [1]. The metabolism of this species and other members of the genus with the same issue has been analyzed by genome-scale metabolic models (GEMs). Some studies have offered a deeper understanding of the metabolic characteristics linked to resistance and have identified potential new targets for antifungal treatment in the most significant *Candida* species [30]. One example is the *FKS/GSC/GSL* genes, which encode the enzyme beta-1,3-glucan synthase and is needed for cell wall synthesis and is considered an essential gene in the *C. albicans* metabolic model named iRV781. Therefore, iRV781 indicates that the enzyme reaction is crucial for the organism’s survival, making it a promising antimicrobial target for this group of microorganisms. Moreover, other antimicrobial molecules, such as ethionamide, sulfacetamide, azelaic acid, cerulenin, or trimethoprim, can act as antimicrobial molecules against some target proteins whose homologous genes have been predicted in the *C. albicans* model. Some examples of those genes are *FAS1*, *TRR1*, and *DFR1*, which are codified to the fatty acid synthase subunit beta [38], thioredoxin reductase [39], and dihydrofolate reductase [40] and have been used as a treatment to other microorganisms, not only for fungal but also for bacteria [30]. As a result, despite the suggested candidates having not been tested in vitro yet, the results from this model could be applied and evaluated to other pathogenic *Candida* species, such as *C. albicans* [30]. Moreover, in the metabolic model of *C. auris*, around 50 essential genes have been predicted as new therapeutic targets for yeast. These genes are unique to this yeast, making their widespread applicability difficult. Indeed, despite the human homologs of this gene having not been described, the treatment’s adverse effects remain unknown. Therefore, more studies are needed to validate these candidates’ efficacy and safety in clinical settings and to analyze potential antimicrobial resistance from the prolonged use of new treatments [33]. Consequently, metabolic models of fungal pathogens stand out as a fresh and effective tool for predicting metabolic targets to advance the search for new antifungal molecules.

Additionally, these metabolic models helped us guess how this species would react to different environmental conditions and nutrient sources. That helped us learn how this microorganism changes to survive in various conditions when they are in an infection process such as the *A. fumigatus* and the dendritic cell GEM. Using the infection process as a reference, the GEM can help us understand *A. fumigatus* and the interaction of human dendritic cells [28]. An increase in the expression of genes such as 4-aminobutyrate transaminase (*GatA*) and alcohol dehydrogenases (*ALDH2* and *ADH*) during DC infection indicates an adaptative behavior of the fungal pathogen to metabolize compounds in the hostile host environment. Likewise, a decreased expression of alkaline phosphatase, branched-chain amino acid aminotransferase (*BCAAA*), and GTP cyclohydrolase I (*GTPCH*) genes suggests metabolic adjustments to immune system aggression [28]. This fungal pathogen has also converted phosphoenolpyruvate into glucose instead of pyruvate when it senses that it does not have enough nutrients. This means that substrate availability changes, and essential amino acids like valine, leucine, and isoleucine are broken down to be used by the pathogen to grow and proliferate when these amino acids are not found in large amounts in the host [28]. However, it is crucial to consider that these models must be accurate at guessing how microorganisms will react to specific conditions to help study the complicated and important cases of infection. This needs to guess could constrain the models as GEMs may not always accurately forecast microbial behavior in all situations. As a result, the model will need to be improved even more to fit as many of the microorganism’s traits as possible, while still meeting the constraints of addiction, enzyme conception, and thermodynamic limitations [41]. As a result, developing GEMs that ensure a highly accurate prediction of the metabolic behavior of the organisms and even expand these findings to include additional pathogenic microorganisms will be required in the future.

Another significant advantage of GEMs is their capacity to examine the interactions between several microorganisms and their host. These models can evaluate bacterial and fungal species, including pathogens and organisms in the microbiota [11,43]. Some studies have investigated several types of symbiosis, with parasitism and commensalism being the most prevalent observed. Even though this has not been examined in terms of fungal pathogens and host interaction, a great example is AGORA (assembly of gut organisms through reconstruction and analysis). This tool is partly automated and allowed us to create 773 GEM strains of bacteria that make up the human intestinal microbiota. These models were used to analyze the microbiota, focusing on predicting and comparing the frequent interactions between microbiota members in co-growth and monoculture. One of the most important results is that the simulations show that some microbiota members grow more slowly in co-cultivation than in monoculture simulations (parasitism). Additionally, the microbiome of certain members grows faster in the paired simulations. In contrast, others were unaffected (commensalism) when the simulations were conducted in different conditions, such as the presence of nutrients due to diet variation or the presence and lack of oxygen [43]. Therefore, this tool has been suggested as the foundation for investigating the interaction between microorganisms and hosts at the gastrointestinal level [44]. However, when studying the relationship between a host and a pathogen, these models allow us to analyze the metabolism of two distinct yet closely related organisms. Constraint-based models have been suggested as a viable method for modeling the interaction between hosts and fungal pathogens. However, at present, these models are only applicable in the study of certain parasites and bacteria.

Moreover, another important application of metabolic models is the incorporation of intricate and diverse data into developing a unified system that represents the interaction between a pathogen and its host; specifically, secondary metabolism takes a principal role. Therefore, various approaches have been employed to examine these secondary metabolites. An instance of this is antiSMASH, the pioneering pipeline capable of identifying loci encompassing all recognized categories of secondary metabolite chemicals in the metabolic processes of bacteria and fungi, utilizing genomic data [44]. This tool has the capability to detect all recognized categories of biosynthetic gene clusters for secondary metabolites, distinguishing it from previous methodologies that were limited to certain types of secondary metabolites [44]. However, different authors agree that existing methods are insufficient in comprehensively capturing the secondary metabolic pathways in genomic metabolic models. This complicates the development of accurate solutions for synthesizing these metabolites [45,46,47]. Therefore, it is imperative to make significant advancements in the tools and approaches to effectively tackle this problem and to broaden the scope of research in this domain to enhance the compression of these intricate yet very advantageous biological systems in studying fungal pathogens and their hosts.

On the other hand, one of the significant limitations of metabolic models constructed for fungi is the reduced information regarding their metabolism. That, combined with the highly intricate and difficult-to-model organisms, has necessitated the comparison of some models with GEMs from other organisms with different characteristics to validate their results. For example, the first models of *S. cerevisiae* metabolism were compared to those of *E. coli* [14]. Another example is using *Ustilago maydis* metabolic and genomic information to make metabolic models for *Malassezia* species [20]. Furthermore, a limited number of studies currently use GEM to analyze metabolic versatility, even more so in fungal pathogens. More specifically, metabolic models of *Malassezia* species have shown how metabolically flexible this yeast species is, especially when it comes to lipid metabolism, which is complicated but interestingly versatile, letting them live and grow even though they cannot synthesize fatty acids, mainly unsaturated fatty acids [20]. As a result, it will be crucial in the future to create models of the organism modeled using omics data, as other authors have suggested, to improve our understanding of the complex organism under study.

In conclusion, metabolic reconstructions have been demonstrated to help investigate human fungal pathogens; however, their application is still in the early stages. Technological advancements, such as machine learning and other established methods commonly used in bacteria but not yet studied in fungi, can greatly improve our knowledge of fungal metabolism. These advancements can lead to new strategies for controlling and treating these organisms. To effectively utilize the potential of metabolic reconstructions in combating fungal infections, it is crucial to emphasize research in the field of computational biology that allows us to improve our understanding of fungal metabolism. In the following section, we discuss some of those newly discovered strategies to analyze fungal metabolism and some other bioinformatics tools used for procaryotic pathogen GEMs and whose use could be efficiently implemented in fungal pathogens.

## 4. Exploring the Future: Multi-Scale Metabolic Models and Machine Learning Strategies

**Figure 2:**
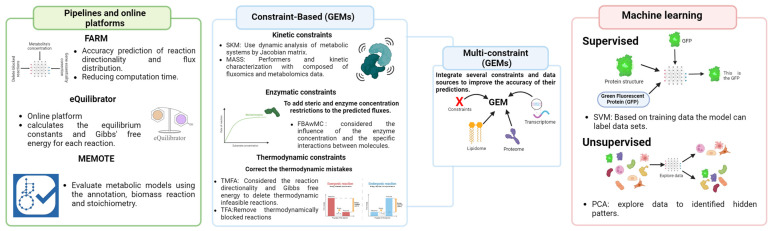
The new computational approaches to performance and high-quality genome-scale metabolic model (GEM). Created with BioRender.com [42].

All the models previously mentioned in this paper have demonstrated some limitations in providing an accurate model for organisms. Also, the biological systems that need to be reconstructed are becoming more complicated, and new ways have been suggested to make it easier to build more advanced GEMs [48]. Scientists have also investigated how to use genome-scale experimental data integration [49] and machine learning tools to make metabolic model predictions more accurate [41]. Therefore, the next part of this review discusses these new methods and the computer programs that make studying the metabolism of microbes that cause fungal diseases easier. It also shows some examples of how they work in the GEM of very different organisms. Figure 2 shows the principal new approaches to creating more accurate genome-scale metabolic models, which should be used in the construction and improvement of those models for fungi organisms.

The bottom-up and top-down approaches represent two different strategies for reconstructing metabolic models at the genome scale. The bottom-up approach relies on the manual construction of models from specific genomic data, which allows for detailed control and in-depth validation, but it is much more laborious and less scalable, especially for studies of microbial communities. On the other hand, the top-down approach uses a universal metabolic model as an initial template, which is automatically adapted through a “carving” process, allowing a fast and efficient generation of specific models without constant manual intervention. The latter approach is the most current as it offers significant advantages in automatization, workload reduction, and scalability, facilitating the modeling of multiple species and communities [50]. One example is the CarveMe pipeline, a Python-based program for genome-scale metabolic model (GEM) reconstruction. This pipeline has a scoring mechanism that connects the similarity of sequences by the gene-protein relations. This strategy enables the removal of responses with insufficient genetic evidence, enhancing the quality of the generated models. CarveMe also allows for the development of consensus models, which integrate genetic and metabolic similarities to improve the functional representation of communities [51]. One significant application of CarveMe 1.6.0 is the GEM of *Saccharomyces cerevisiae* named Yeast8 (https://github.com/SysBioChalmers/proYeast8-GEM, accessed on 6 October 2024) [25], and CarveFungi (https://github.com/SandraCastilloPriego/CarveFungi, accessed on 6 October 2024), another Python-based tool that can be integrated into CarveMe for automatic compartmentalization [52]. Moreover, CarveMe has integrated supplementary software tools to enhance the results of its GEMs as the Genome-scale model to account for Enzyme Constraints GECKO 3.0, a powerful metabolic modeling tool that enables the construction and examination of genome-scale metabolic networks. GECKO integrates genetic, environmental, and gene expression data for microorganisms such as yeasts [25]. This tool primarily focuses on metabolic kinetics, enabling researchers to gain insights into the reactions that can take place and their behavior under different conditions. Moreover, this helps to predict the metabolic fluxes, enhances metabolic pathways to increase the production of desired metabolites, and evaluates the performance of pathways in response to external stimuli. This makes it extremely helpful for applications in the fields of biotechnology and synthetic biology [53]. It is not surprising that this computational tool will be used in the future to develop new GEMs of fungal organisms, both pathogenic and non-pathogenic, as well as to enhance already-existing models.

Furthermore, the 2020 publication of the metabolic model testing suite MEMOTE 0.14.0, a bioinformatic tool, provides several advantages that significantly enhance the effectiveness of model comparisons and validations. This enables us to assess the quality of GEM by testing the fundamental areas of GEM reconstruction, such as the annotation steps, the biomass reaction, and the model’s stoichiometry [54]. This tool can help users evaluate the model’s acceptance standards, verify its formal accuracy, and ensure consistency in biomass generation and stoichiometry. The software produces detailed reports that quantify the model’s performance in a manner easily understood by the user. Additionally, the software is compatible with various platforms, such as GitHub, GitLab, and BioModels, enhancing its accessibility [54]. Therefore, it is not surprising that future metabolic models for the GEM of fungal pathogens will employ this pipeline. Furthermore, as described above, these restriction-based approaches are essential for building models of pathogen–host interaction in biological systems [55].

Changes in the concentration of an enzyme required to carry out a specific biological process are also important considerations. Therefore, Flow Balance Analysis with Molecular Crowding (FBAwMC) could be used to make the GEMs that consider how enzyme concentration and molecule interactions affect the flow of metabolites in a living system. Among the advantages of this methodology is that it creates a more accurate and useful GEM by adding steric restriction and enzyme concentration constraints. Moreover, this considers the physical and chemical limits of the cell environment to provide a better comprehension of the biological system. This computational tool enables a more accurate representation of metabolic processes in GEMs [41]. Although this tool has not been utilized in the GEM of eukaryotes, it has been employed in the past for other pathogenic organisms like *E. coli*. After using it, the predicted growth rate and the experimental results were more accurate. It also helped determine the order in which different substrates would be used because of the limits of enzyme concentration [56]. On the other hand, some models have already integrated enzymatic and kinetic constraints into their construction; however, those two alone are not enough to show how complicated organisms like eukaryotes, especially fungi, are. Moreover, conventional genome-scale metabolic models, constructed with only one set of data from a single gene–protein relationship, still fail to capture the complexity of biological systems fully. Therefore, developing multi-constraint GEMs is seen as a more precise method than conventional GEMs. These models integrate various restrictions or data sources to enhance their predictions’ precision. Multi-constraint GEMs get around this problem by adding information like genomic data, transcriptional regulation networks (TRNs), and protein structure (PRO). That gives us a fuller description of how cells work and their metabolic networks and regulatory mechanisms. By integrating numerous limitations, these models can more accurately forecast phenotypic characteristics using genotypic data [41]. Therefore, although this is not a new tool, in the future, it will be imperative that this type of constraint be used for modeling fungal pathogens, as it has been used for bacteria.

Alternatively, machine learning techniques such as regularized multinomial logistic regression and random forests have been suggested as novel methods to address the constraints of metabolic reconstructions. Moreover, integrating omics data enhances metabolic reconstructions by contextualizing predicted metabolic relationships in the GEM. However, the efficiency of handling and deriving relevant knowledge from vast and complex data sets poses a significant challenge to the incorporation of omics data. Thus, machine learning techniques can be utilized to train tools that can predict metabolic behavior and retrieve significant information with minimal manual work. In this regard, machine learning can be categorized into two main approaches: supervised and unsupervised learning. Supervised learning uses pre-classified data to create a coordinated result. Some proper methods for this are support vector machines (SVMs), which can be used to solve classification and regression problems [57]. In this regard, SVM has been applied to classify species of *Cryptoccocus*, the cause of fatal infections in immunocompromised patients. Moreover, this supervised ML tool has shown highly accurate classification between *C. neoformans* and *C. gatii* with a sensitivity and specificity of 100%. This tool also includes variable selection and reduction methods to work with complex ATR-FTIR data so that different *Cryptococcus* species can be told apart by their unique patterns. Because of this, this tool, which has shown important advantages to fungal pathogen characterization, provides a solid and accurate way to tell the difference between species by utilizing its ability to handle complex data. It is an efficient means for promptly and non-invasively diagnosing severe fungal infections [58], even of other fungal pathogens. On the other hand, the non-supervised learning tools allow the exploration of data collections by evaluating the correlations between analyzed samples. Unsupervised learning techniques enable researchers to identify patterns and group data from samples according to their characteristics, which are frequently impossible for the researcher to infer. Given a large amount of omics data, their grouping or simplification may be helpful in facilitating their interpretation. Principal component analysis (PCA), for example, is particularly useful in the study of metabolism due to the substantial volume of data that needs to be processed, and the complexity of the variables involved [59]. In fungi, machine learning algorithms like this have been employed to analyze some collections of metabolites generated by various species of *Aspergillus* and *Penicillium*, and this approach has allowed us to propose their function as bioactive compounds because antimicrobial molecules demonstrated their usefulness in the analysis of therapeutic targets against different human pathogen [59].

Furthermore, the study of microbial community data can utilize the newly developed non-negative matrix factorization (NMF) strategies, which offer numerous advantages. A good example of this is the triUMPF (triple non-negative matrix factorization [NMF] with community detection for metabolic pathway inference), which combines three stages of NMF and can capture numerous relationships between pathways and enzymes, all of which are integrated into a graph network [60]. Identifying microbial subcommunities, composed of microorganisms that are functionally dependent on one another, is one of this tool’s advancements, which is essential to comprehending the composition and roles of microbial communities. NMF also looks at different kinds of data, like metagenomic and OTU data, showing that it can be changed and adapted to meet the needs of various organisms and the microbial communities underneath them. Furthermore, this machine learning method makes it easier to understand the biological meaning of the results by revealing patterns in the data that were not identified before and improving the ability of organisms to be classified into the subcommunities found [61]. Therefore, given the close relationship that must be established between them, it should not be ruled out that this tool will be helpful in the future for the analysis of interactions between fungal pathogens and their hosts, as well as in the study of multifactorial diseases and to understand the role of the various microorganisms that make up the microbiota, because it is already known that some pathologies develop as a result of imbalances in the dynamics of microbial communities rather than as a result of single microorganism colonization [43]. In this regard, the metagenome-scale modeling of gut microbiota (MICOM) is an advanced bioinformatic tool specifically developed to simulate metabolic interactions within microbial communities accurately. The primary purpose of this tool is to enable researchers to construct comprehensive mechanistic theories regarding the interactions between various microbiota member species and their environment [62]. This tool combines metagenomic data, dietary information, and genome-scale metabolic models, making it possible to make predictions about how microbial communities interact with their environment. Moreover, this offers a beneficial structure for investigating the metabolic pathways that underlie the states of health and sickness in the host and for understanding the intricate relationships within microbial communities and improving interventions in the microbiota to enhance human health, an important element that can influence fungal infection development [62]. Therefore, in the future, we should use these approaches to incorporate multiple data sources into the analysis of microbial metabolism, especially the metabolism of fungal pathogens, since, as we have described earlier, their implementation allows us to generate more accurate GEMs to the actual context of this pathogen of high importance for humans.

Here, we present a comprehensive overview of genome-scale metabolic models (GEMs) for fungal pathogens, highlighting their potential for understanding fungal metabolism and developing therapeutic strategies. However, some limitations are evident within this work. Other authors emphasize specific issues that should have been more extensively covered in this review. For instance, Tarzi et al. 2024 [63] discuss the difficulties of verifying community models, emphasizing the need for more efficient computing methods and standardized protocols for the GEMs of microbial communities’ analysis. Moreover, despite the promise of integrating contextual data, a significant gap persists in the research required to implement these methodologies in microbial communities effectively. In this regard, it will be crucial to consider these constraints when examining the interaction of microbial communities and the metabolism of its members. However, with this review, we encourage other authors to advance the development of new computational tools to overcome these difficulties. Moreover, according to Demangel and Surace, 2024 [64], more research needs to be carried out on how certain metabolites might affect the host’s immune responses. They discuss that integrating these approaches into a cohesive understanding of immunometabolism is a complex task that necessitates ongoing interdisciplinary collaboration and innovation. We could not agree more with that, and we remain optimistic that this review will promote the interdisciplinary integration of various expert groups in microbiology and systems biology. With that, we can investigate the various interactions that have developed in infections brought on by fungal pathogens.

## 5. Conclusions

Applying metabolic models at the genome scale has significantly revolutionized the study of microbial metabolism. Metabolic studies have transformed a list of reactions and metabolites as genome-scale network reconstructions into intricate mathematical models that can precisely forecast the activities of more complex organisms. Nevertheless, only a few examples of these models are employed to analyze the pathogenicity of human fungal diseases. Furthermore, additional technologies are continuously being incorporated into creating new and more complex models that are more closely aligned with reality. Integrating these novel methodologies into fungal metabolic models will be essential to advancing our understanding of fungal infections at the metabolic level.
